# MammOnc-DB, an integrative breast cancer data analysis platform for target discovery

**DOI:** 10.21203/rs.3.rs-4926362/v1

**Published:** 2024-09-26

**Authors:** Sooryanarayana Varambally, Santhosh Kumar Karthikeyan, Darshan Chandrashekar, Snigdha Sahai, Sadeep Shrestha, Ritu Aneja, Rajesh Singh, Celina Kleer, Sidharth Kumar, Zhaohui Qin, Harikrishna Nakshatri, Upender Manne, Chad Creighton

**Affiliations:** University of Alabama at Birmingham; University of Alabama at Birmingham; University of Alabama at Birmingham; University of Alabama at Birmingham; University of Alabama at Birmingham; UAB; Morehouse School of Medicine; University of Michigan; University of Illinois Chicago; Emory University; Indiana University - Indianapolis; University of Alabama at Birmingham; Baylor College of Medicine

## Abstract

Breast cancer (BCa) is one of the most common malignancies among women worldwide. It is a complex disease that is characterized by morphological and molecular heterogeneity. In the early stages of the disease, most BCa cases are treatable, particularly hormone receptor-positive and HER2-positive tumors. Unfortunately, triple-negative BCa and metastases to distant organs are largely untreatable with current medical interventions. Recent advances in sequencing and proteomic technologies have improved our understanding of the molecular changes that occur during breast cancer initiation and progression. In this era of precision medicine, researchers and clinicians aim to identify subclass-specific BCa biomarkers and develop new targets and drugs to guide treatment. Although vast amounts of omics data including single cell sequencing data, can be accessed through public repositories, there is a lack of user-friendly platforms that integrate information from multiple studies. Thus, to meet the need for a simple yet effective and integrative BCa tool for multi-omics data analysis and visualization, we developed a comprehensive BCa data analysis platform called MammOnc-DB (http://resource.path.uab.edu/MammOnc-Home.html), comprising data from more than 20,000 BCa samples. MammOnc-DB was developed to provide a unique resource for hypothesis generation and testing, as well as for the discovery of biomarkers and therapeutic targets. The platform also provides pre- and post-treatment data, which can help users identify treatment resistance markers and patient groups that may benefit from combination therapy.

## 1. Introduction

Breast cancer (BCa) is one of the most common cancers in women worldwide. Since the mid-2000s, the incidence of BCa has increased by approximately 0.5% annually. ^1^ The etiology of BCa involves factors such as genetic predisposition, lifestyle changes, and aging ^2^. Genetic mutations, familial history, demographic variables, medical background, and modifiable risk factors such as obesity, alcohol intake, and smoking are involved in its development ^3–5^. BCa tumors are classified into distinct subtypes (Luminal A, Luminal B, HER2+, and TNBC), characterized by expression levels of estrogen and progesterone receptors, and HER2 expression in tumor cells. The hormone receptor-expressing BCa as well as HER2-positive tumors have viable treatment options ^4,6^. Early-stage BCa is considered curable; however, despite significant progress in diagnosis and treatment, advanced/metastatic stage is associated with high mortality. Although BCa initially responds to treatments, may eventually, can recur and develop therapy resistance ^7,8^. However, the heterogeneity of BCa poses a substantial challenge in diagnosis and treatment, requiring precision medicine to address the diverse molecular subtypes involved^9^.

With the availability of high-throughput technologies from advanced molecular profiling, such as next-generation sequencing and mass spectrometry, researchers can evaluate specific biomarkers and molecular signatures associated with tumor subtypes and identify potential therapeutic targets ^10^. Although data from next-generation sequencing have shed light on the molecular evolution of BCa, it is necessary to understand and process these molecular data with clinical information to enhance the capability of precision medicine and precision targeting approaches ^11^. Although large amounts of data are available in public repositories, there are opportunities to develop user-friendly resources that allow cancer researchers to leverage the data effectively.

Large-scale cancer “Omics” data, generated using various techniques such as microarray, bulk RNA-seq, scRNA-seq, ChIP-seq, ATAC-seq, and MS/MS data for genetic, epigenetic, and proteomic data, are archived in numerous public repositories. From the perspective of a researcher with limited bioinformatics support, performing an in-depth analysis of the volume of genomic and proteomic data available for BCa is challenging. A focused and comprehensive web resource that provides integrative analysis, including data for metastatic BCa and response to BCa treatments, will be useful. Recognizing unmet need and opportunities to develop a comprehensive resource facilitating BCa data analysis and visualization, we developed the MammOnc-DB, a user-friendly portal for integrative analysis and visualization of BCa data.

MammOnc-DB incorporates data that were collected, curated, and integrated from the NCBI Gene Expression Omnibus. In addition, we utilized Proteomics Identifications Database (PRIDE) and ProteomeXchange to obtain proteomic data. MammOnc-DB also contains multi-omics data from The Cancer Genome Atlas (TCGA), Clinical Proteomic Tumor Analysis Consortium (CPTAC), the METABRIC, Cancer Cell Line Encyclopedia (CCLE), and Sweden Cancerome Analysis Network – Breast (SCAN-B) Consortium. Our data procurement and processing included multiomics studies that included data for normal breast tissue, primary BCa tissue, and metastatic BCa samples, with associated clinical information. In addition, we included data on BCa patients treated with various therapies.

Using MammOnc-DB, researchers can access multi-omic and multiple publicly available BCa datasets. It provides information and enables users to analyze the expression of genes (mRNAs, miRNAs, and lncRNAs) and proteins in primary and metastatic BCa along with available normal samples and across tumor subgroups based on tumor stage, tumor grade, race, molecular subtype, histological subtype, or other available clinicopathologic features. By utilizing MammOnc-DB to identify differentially expressed genes, one can identify the top differentially expressed genes associated with specific clinical features. Additional options include Kaplan-Meier survival analysis and evaluation of epigenetic changes. Users can download high-resolution graphics depicting expression profiles and patient survival information in various forms.

The MammOnc-DB enables researchers to utilize high-throughput BCa omics data to identify potential biomarkers and therapeutic targets for BCa. Furthermore, *in silico* validation of selected genes using the independent studies integrated into this platform. With subgroup-specific data analysis, one can identify gene alterations in subsets of BCa, allowing the development of hypotheses and testing the underlying biology for this dysregulation. In the future, our goal is to populate the MammOnc-DB platform with additional data as they become available.

## 2. Methods

### 2.1. Data Collection and Analysis:

#### 2.1.1 TCGA, CPTAC, SCAN-B, METABRIC, and CCLE:

The Cancer Genome Atlas (TCGA) provides data on genomics and transcriptomics for various cancers. We downloaded RNA-sequencing data from Genomics Data Commons (https://portal.gdc.cancer.gov/) related to TCGA Breast cancer (BRCA). As TCGA provided level-3 data, we did not perform data processing. In addition, we downloaded methylation data from TCGA BRCA using the DownloadMethylationData() function from TCGA-assembler (https://ccte.uchicago.edu/TCGA-Assembler/index.php). The unwanted column information in the data was removed by using ProcessMethylation450Data(). When CpG sites corresponded to more than one gene, average methylation values were calculated using CalculateSingleValueMethylationData().

We also obtained processed transcriptomic data from studies such as SCAN-B, ABiM_405, ABiM_100, OSLO2-EMIT0 ^12–15^, Creighton Breast Tumor Compendium^16,17^, Van de Vijver *et al.*^18^, Neo-adjuvant Chemotherapy Response Compendium dataset ^19^, and METABRIC dataset^20^ through literature search. These studies included gene expression values along with the patient clinical features.

From the Human Cancer Cell Line Encyclopedia (CCLE) and DepMap portal (https://depmap.org/portal/download/all/), CRISPR knockout screens of BCa cell lines were obtained as gene-effect scores from Achilles and Sanger’s SCORE project. In this study, the scores were normalized so that nonessential genes had a median score of 0, while independently identified common essential genes have a median score of −1. Gene Effect scores were inferred using Chronos^21^. The integration of the Broad and Sanger datasets followed the methodology outlined by Pacini et al., with the exception that quantile normalization was omitted^22^.

In addition, we downloaded the BCa proteomics data from Clinical Proteomic Tumor Analysis Consortium (CPTAC) from Proteomics Data Commons (https://proteomics.cancer.gov/programs/cptac). The integration and analysis of these data have been previously reported ^23,24^. In summary, protein expression values downloaded from the CPTAC data portal were log2 normalized for each sample. Z-values for each protein in each sample were then calculated as the number of standard deviations from the median across samples.

#### 2.1.2. RNA-seq Data Analysis:

We procured raw data from NCBI GEO for GSE58135 ^25^, GSE142731^26^, GSE183947 ^27^, GSE100925 ^28^, GSE47462 ^29^, GSE184196 (https://www.ncbi.nlm.nih.gov/geo/query/acc.cgi?acc=GSE184196), GSE122630 ^30^, GSE163882 ^31^, GSE130660 ^32^, GSE99063 ^33^, GSE68359 ^34^, and GSE131276^35^.The raw data from NCBI GEO were downloaded using fastq-dump function from SRA Toolkit (https://github.com/ncbi/sra-tools). The adapter sequences in the downloaded fastq files were trimmed and quality checked by Trim Galore (https://github.com/FelixKrueger/TrimGalore). The trimmed files were mapped to hg38 genome by using the HISAT2 (https://daehwankimlab.github.io/hisat2/) alignment tool, followed by bam conversion and sorting by SAMTools ^36^. The gene counts from the bam files were obtained by using HTseq-counts function ^37^. The gene counts were converted either to FPKM or to RPKM by using R or the Python package, respectively (https://github.com/AAlhendi1707/countToFPKM). When raw data were not available for studies such as GSE209998 ^38^, GSE173661 (https://www.ncbi.nlm.nih.gov/geo/query/acc.cgi?acc=GSE173661), and GSE96058 ^15^,we procured the processed data and performed downstream analysis. The statistical analysis was conducted with an unpaired welch t-test.

#### 2.1.3. Gene Expression Array Data Analysis

For the “Creighton Breast Tumor Compendium” dataset^16,17^ of nine separate breast tumor expression profiling datasets for survival analysis, gene transcription profiling datasets (all on Affymetrix U133 array, A set, and all with DMFS as an outcome measure) were obtained from previous studies (Loi, GEO:GSE6532; Wang, GEO:GSE2034; Desmedt, GEO:GSE7390; Miller, GEO:GSE3494; Schmidt, GEO:GSE11121; Zhang, GEO:GSE12093; Minn, GEO:GSE2603 and GEO:GSE5327, Chin, http://cancer.lbl.gov/breastcancer/data.php. Genes within each dataset were first normalized to standard deviations from the median; samples from the Loi dataset that were also represented in Desmedt were excluded from Loi. When multiple gene array probe sets referenced the same gene, the probe set with the highest average variation across samples for the nine datasets was selected to represent the gene.

For the chemotherapy response expression compendium dataset^19^, we previously assembled a compendium of eight different public breast cancer expression datasets^39–45^, involving gene expression profiling of pre-treatment breast tumor biopsies from patients treated with neoadjuvant chemotherapy, with patient response recorded at the end of treatment. The compendium, representing 1240 tumor expression profiles, involved all datasets being generated using the same Affymetrix gene array platform. We normalized the expression values within each dataset in the same manner as described above for the Creighton dataset.

#### 2.1.4. Proteomics Data Analysis Pipeline:

The output files from PRIDE were converted to raw format using msConvert ^46^. We obtained raw files for studies such as PXD012431^47^ and PXD018830 ^48^ from PRIDE. MaxQuant and Andromeda search engines were used to process the downloaded MS/MS data, with reference to *Homo sapiens* UniProt proteome (UP000005640) ^49^. The MaxQuant parameters were set based on the proteolytic enzyme used, fixed and variable modifications, quantification approach, and data acquisition method. To perform downstream statistical analysis, the output files from MaxQuant analysis were used as input files for Perseus ^50^. NA values were eliminated from the resulting file, considering the condition that the row should have only three or fewer values. Additionally, the values were log-normalized for further analysis. In addition, we also downloaded processed gene level proteomics data from Anurag M et al., research article ^51^.

#### 2.1.5. ChIP-seq Data Analysis:

The data associated with GSE85158 ^52^, GSE117941 ^53^, and GSE178373 ^54^ studies were downloaded from NCBI GEO using the fastq-dump from SRAToolkit (https://github.com/ncbi/sra-tools). The quality of the raw data was assessed by FastQC (https://github.com/s-andrews/FastQC), followed by removing the adapter sequences using Trim Galore (https://github.com/FelixKrueger/TrimGalore). The human reference (hg38) was used for alignment with trimmed reads, using BWA mem ^55^. Duplicate reads were identified using Picard (https://github.com/broadinstitute/picard), followed by merging the technical replicates using SAMtools ^36^. The obtained bam files were converted to bed and bigwig files using BamToBed and bamCoverage tools ^56^. Peak calling was performed (NarrowPeaks for transcription factors and Broad Peaks for histone modification) with input DNA or IgG as controls, using MACS2^57^.

#### 2.1.6. scRNA-seq Data Analysis:

The processed data for BCa single-cell sequencing were downloaded from the Curated Cancer Cell Atlas (https://www.weizmann.ac.il/sites/3CA/) ^58^. We procured associated data and meta files for studies byQian *et al* ^59^, Gao *et al* ^60^, Azizi *et al* ^61^, Wu *et al* ^62^, and Griffiths *et al* ^63^. Using the Seurat R package, we filtered the cells to have at least 1000 genes in each barcode ^64^. These filtered cell counts were normalized, batch-corrected using Harmony, and annotated based on the available clinical features^65^.

### 2.2. Data formatting and visualization:

We integrated genomic, proteomic, and epigenetic studies into a user-friendly web resource built using PERL CGI. The data analysis results were depicted via interactive visualizations using public and in-house Java script libraries, and Python Flask applications.

Using R and PERL scripts, gene expression matrix files from RNA-seq and scRNA-seq studies and protein expression matrix files from proteomic studies were categorized based on tumor grade, tumor stage, patient’s age, patient’s race, nodal metastasis status, molecular subtype, treatment, and other associated categories.

Categorized and formatted data files were utilized to generate various graphical outputs such as heatmaps, box plots, jitter plots, Kaplan-Meier curves, UMAP plots, and violin plots as representations that address heterogeneity by comparing gene/protein expression along with various clinical features in each dataset.

ChIP-seq results highlighting epigenetic modifications near the gene region are displayed as IGV plots.

#### 2.2.1. Visualization of differentially expressed genes:

Heatmapvisualization was employed to visualize the most differentially expressed mRNAs, miRNAs, lncRNAs, and proteins in various BCa datasets. To compile a list of the top 250 genes that exhibited either over-expression or under-expression in each subtype, we initially identified genes with FPKM values that displayed significant differences (p-values < 0.05). From this initial selection, we considered only genes with a median FPKM value of 1 or higher. Finally, the genes were ranked based on the ratio of the mean FPKM values in tumor samples to the mean FPKM values in normal samples. To generate an interactive heatmap illustrating the top over- and under-expressed genes in a dataset, we utilized the Highcharts library from JavaScript (http://www.highcharts.com/).

#### 2.2.2. Visualization of individual gene expression patterns:

Box and Jitter plots were employed to depict the expression levels of the genes in normal samples, primary breast tumors, metastatic breast tumors, and various treatment groups, along with the associated clinical characteristics. The Highcharts library from JavaScript was used to generate the visualizations representing the interquartile range (IQR), including minimum, 25th percentile, median, 75th percentile, and maximum values, utilizing the data obtained from data formatting.

#### 2.2.3. Visualization of scRNA-seq based gene expression:

The techniques utilized for visualizing single-cell RNA-seq data included UMAP, violin plots, and ridge plots. These visualizations were generated using Python, with pandas (https://pandas.pydata.org/) for data manipulation and Plotly (https://plotly.com/python/) for creating the plots. This approach allowed the display of gene expression patterns across various cell types and the representation of clustering outcomes. The resulting images were stored and presented through HTML embedding, allowing for interactive exploration and analysis of the single-cell RNA sequencing data.

#### 2.2.4. Survival analysis using Kaplan-Meier curves:

Patient survival data and gene or protein expression data from each dataset were utilized to create Kaplan-Meier survival plots. A Perl script developed in-house was employed to generate input files for survival analysis, which included details such as patient id, survival time (days/months), patient vital status (alive or deceased), and sample categories such as high-expression and low/medium-expression groups. Patient categorization for survival analysis was performed as previously described in Chandrashekar et al ^66^. To conduct multivariate analyses, clinical features such as race, sex, subtype, and grade, among others, were considered in relation to the expression and survival information. The “survival” and “survminer” packages in R were utilized for univariate and multivariate survival analyses, and statistical significance was assessed using log-rank tests (https://cran.r-project.org/web/packages/survminer/index.html). Finally, in-house JavaScript Kaplan-Meier plots were created for genes in the dataset for which survival information was available.

#### 2.2.5. Visualization of ChIP-seq data:

To facilitate the interactive visualization of data from ChIP-seq analysis, the MammOnc-DB platform incorporated the “igv.js” JavaScript developed by the IGV team (https://github.com/igvteam/igv.js/) for peak calling. Bigwig files and broadpeak/narrowpeak files from ChIP-seq data analysis were loaded to igv.js to generate IGV plots.

### 2.3. Web server Configuration:

MammOnc-DB operates on a CentOS server that has 72 cores (Intel^®^ Xeon^®^ CPU E2–2699 v3 @ 2.30GHz), 98 GB of RAM, and 22 TB HDD. To provide users with a seamless experience, the user interface of MammOnc-DB was created using PERL-CGI hosted on the Apaches webserver (https://httpd.apache.org/).

## 3. Results

### 3.1. Overview:

**Figure 1:**
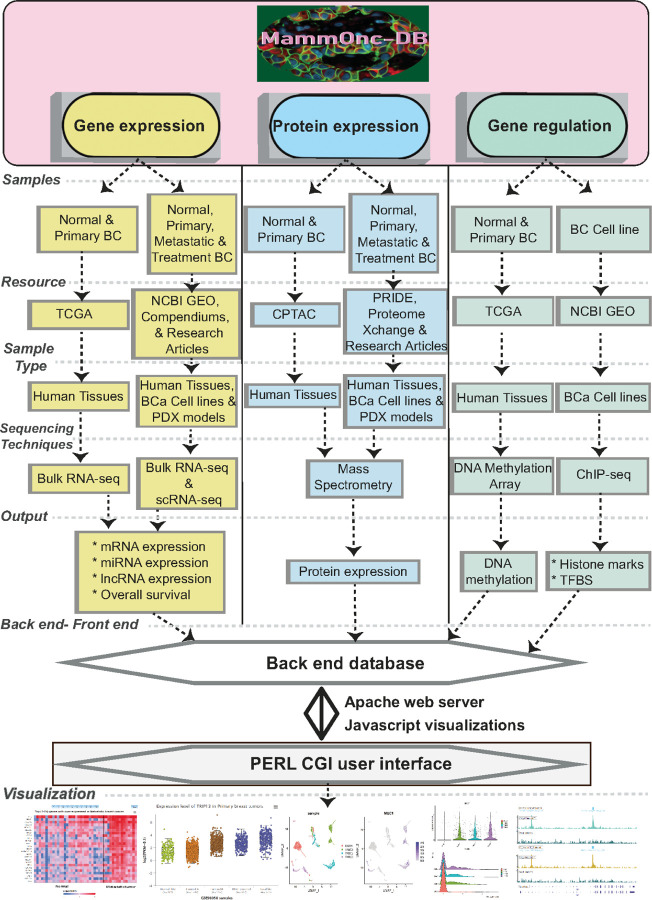
Graphical Abstract. MammOnc-DB, a web-based proteo-genomics platform for analysis and visualization of multi-omics breast cancer data.

Figure 1 provides an overview of MammOnc-DB, and Supplementary Table 1 lists the currently available studies within the MammOnc-DB.

The MammOnc-DB homepage allows users to select the type of omics they are interested in, such as gene expression, protein expression, and gene regulation, through the menu bar. Additionally, the platform also contains a tutorial page to assist users in using the portal effectively

The functionality of MammOnc-DB extends to various types of analysis, which are described in the following sections.

### 3.2. Heatmap facilitating identification of top differentially expressed genes.

**Figure 2:**
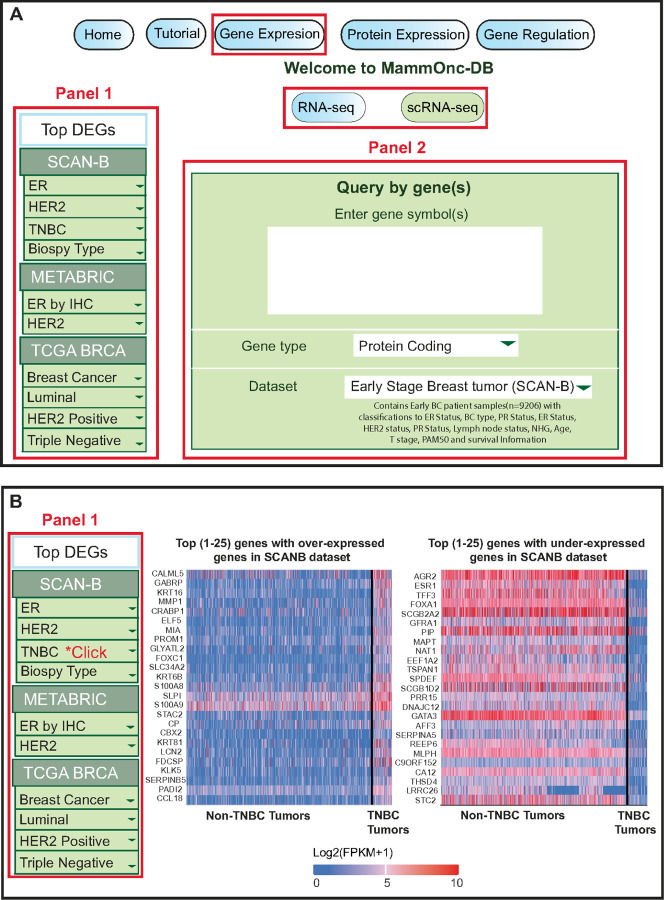
An overview of Gene Expression analysis. (A) Users can switch between RNA-seq and scRNA-seq data. In Panel 1, users can access a compilation of studies, along with relevant clinical characteristics, allowing for the examination of over-expressed and under-expressed genes. Panel 2 allows users to assess the expression of genes of interest across various studies. **(B)** Heatmap generated from Panel 1 of the gene expressionpage. The Heatmap shows the top over-expressed and under-expressed genes in the SCAN-B dataset, comparing non-TNBC and TNBC tumors.

The gene expression page of MammOnc-DB features a left panel that allows users to identify genes that are either over or under-expressed in a dataset (Fig. 2A). For instance, if a user selects “TNBC” under “SCAN-B” in Panel 1, they will be directed to a dedicated page that displays the over-expressed and under-expressed genes in the form of a heatmap. Figure 2B shows a heatmap representing the top 25 genes that are over- or under-expressed, comparing non-TNBC tumors (n = 8332) and TNBC (n = 874) tumors in the SCAN-B dataset. This page allows users to identify up to the top 250 over-or under-expressed genes in the dataset. Moreover, by clicking on the gene name in the chosen study, users can access expression information about each gene in that study. Additionally, our portal offers the option of identifying over and under-expressed lncRNAs and miRNAs using heatmap (Supplementary Fig. 1).

### 3.3. Identifying the expression pattern of a queried gene across different datasets with subgroup classifications:

#### 3.3.1. Overview of gene expression and survival analysis using bulk RNA-seq and microarray datasets:

**Figure 3:**
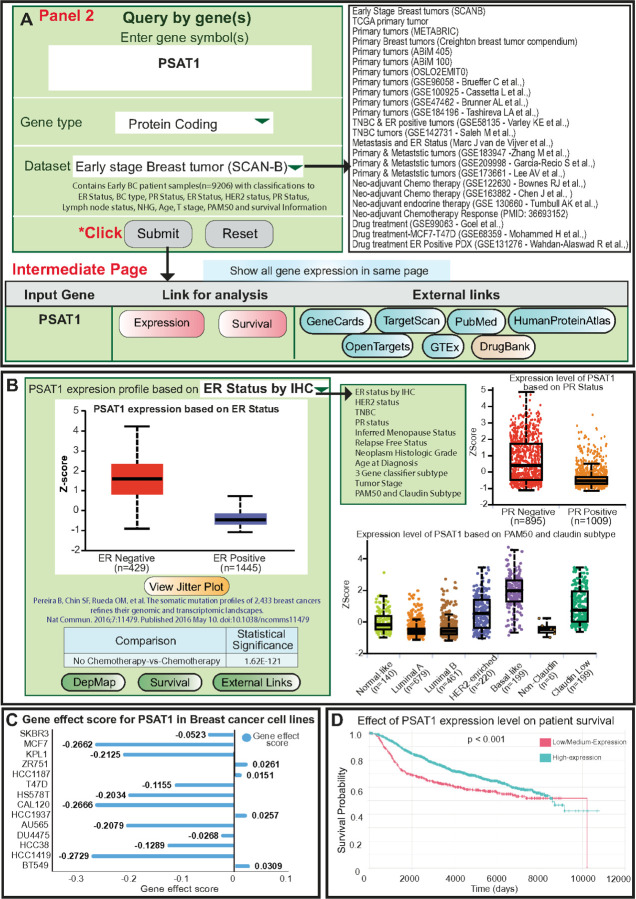
Overview of gene exploration across various studies. (A) Users can explore genes of interest by entering their names into the text box and selecting from available studies. Upon submission, users are redirected to an intermediate page listing links to analyze expression and survival associations. (B) Box-whisker and jitter plots illustrating *PSAT1* expression in subgroups of the METABRIC study, including ER Status, PR Status, and PAM50 and Claudin subtypes, and lists additional available classifications. (C) Bar plot depicting the gene effect score of *PSAT1*in multiple breast cancer cells using data from DepMap. (D) Kaplan-Meier plots showing the association between PSAT1 expression and patient survival in the METABRIC dataset.

Using Panel 2 on the gene expression page, users can search for their specific gene of interest and determine whether it is related to protein-coding, miRNA, or lncRNA across a range of datasets and analyze their expression patterns in relation to various clinicopathologic features (Fig. 2A). In the gene expression page, users have the option to select between “bulk RNA-sequencing” or “scRNA-seq” data, enabling them to input their gene of interest and choose a study from the available choices (Fig. 3A). MammOnc-DB currently offers 20 studies for bulk RNA-seq (TCGA-BRCA, SCAN-B, ABiM_405, ABiM_100, OSLO2EMIT0, GSE58135, GSE142731, GSE183947, GSE100925, GSE47462, GSE184196, GSE122630, GSE163882, GSE130660, GSE99630, GSE68359, GSE131276, GSE209998, GSE173661,and GSE96058), two microarray (METABRIC and Van de Vijver et al.), two microarray compendium datasets (Creighton breast tumor compendium and Neo-adjuvant chemotherapy compendium), and five scRNA-seq studies (Qian et al., Gao et al., Wu et al., Azizi et al., and Griffiths et al.,), which are categorized into primary, metastatic, and treatment-related studies of BCa.

For example, the *PSAT1* gene was typed in the text box, “protein-coding” was the gene type and the “METABRIC” study was selected. Clicking the “Submit” button leads them to an intermediate page displaying the gene name, analysis types, and external links to additional resources (Fig. 3A). Clicking on the “Expression” button directs users to the expression page, where box and jitter plots with corresponding p-values for various categories are presented, with the statistical analysis being an unpaired Welch t-test. Figure 3B shows a boxplot that illustrates the expression pattern of *PSAT1* in the METABRIC study. It compares ER Negative (n = 429) and ER (n = 1445), positive patients, showing a statistically significance with a p-value less than 0.001. Users can also visualize the results in terms of jitter plots by clicking the button. Examples of *PSAT1* expression in METABRIC, based on PR Status, and PAM50 and Claudin subtype are shown as jitter plots in Fig. 3B. Additional studies and classifications for different genes are represented in Supplementary Fig. 2.

The DepMap button at the bottom allows users to access a comprehensive dataset consisting of 40 BCa cell lines and their corresponding gene effect scores. These scores are derived from CRISPR knockout screens conducted by Dempster *et al.*^21^ This feature allows users to assess the impact of gene knockout in each cell line. An example of *PSAT1* gene knockout and the associated gene effect score in various breast cancer cell lines are depicted as a bar plot in Fig. 3C.

In addition to analyzing gene expression, users can utilize the “Survival” button to perform Kaplan-Meier analysis for their genes of interest. The survival profile of *PSAT1* in the METABRIC dataset shows that higher expression of *PSAT1* was significantly associated with poor survival (p < 0.001), as illustrated in Fig. 3D. Supplementary Figs. 3A and 3B present additional multivariate Kaplan-Meier plots of lncRNA (*PCAT1*) and miRNA (*hsa-mir-7706*) from TCGA dataset.

#### 3.3.2. Single-cell *RNA-seq* data analysis:

**Figure 4:**
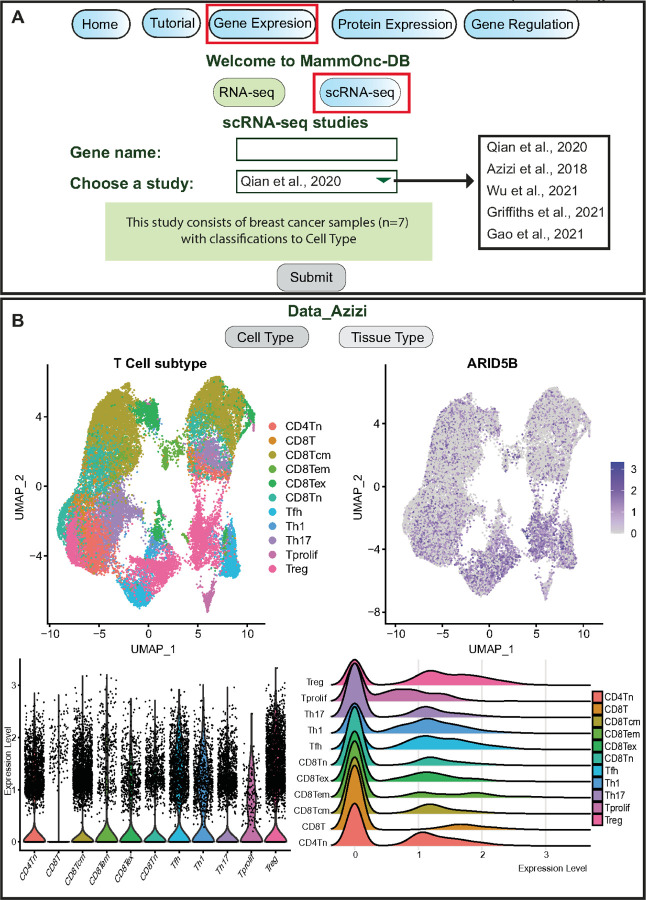
Illustration of the single cell RNA-seq data analysis functionalities. (A) Users can input a gene of interest and select from available studies. (B) Expression of *ARID5B*across various T cell clusters from Azizi et al. (2018) study. The expression is visualized using UMAP, violin plot, and ridge plot, providing insights into the gene’s expression patterns in distinct T cell clusters.

Furthermore, users can retrieve scRNA-seq data through the gene expression section, allowing them to discern expression patterns within various clusters visualized as UMAP, violin plots, and ridge plots (see Fig. 4). An illustration of the expression pattern of *ARID5B* in Azizi et al., is provided as an example, displaying UMAP, violin plots, and ridge plots, comparing its expression in different subclasses of T cells. Additional studies and classifications are presented in Supplementary Fig. 4.

### 3.4. Analyzing the expression patterns of target proteins across various datasets and patient subgroups

**Figure 5:**
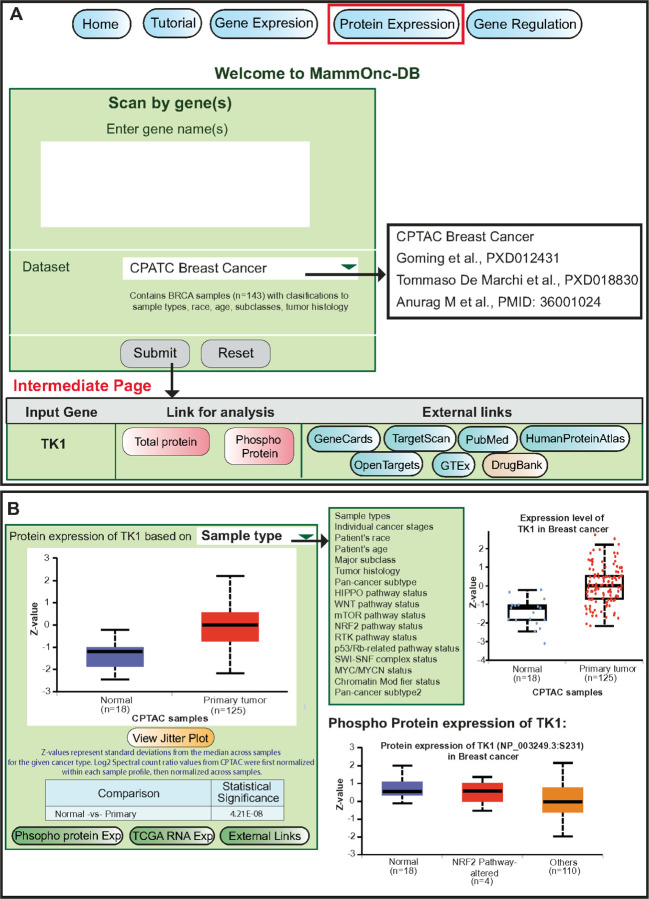
Protein expression analysis in MammOnc-DB. (A) Users can input a gene of interest and perform various analysis from the available studies. (B) Expression pattern of TK1 total and phospho-protein are shown as an example in various clinical features available in CPTAC.

Users can determine the expression pattern of a specific protein by utilizing the protein expression page in MammOnc-DB. This page was designed similarly to the gene expression page. Users can input the name of the gene of interest for the available studies (CPTAC, Tommaso De Marchi et al., (PXD01431), Goming et al., (PXD018830), and Anurag M *et al.,*) and the protein expression results were observed through a box and jitter plot format (Fig. 5A). An illustrative example of TK1 expression is shown in Fig. 5B, which displays the total and phosphoprotein expression of TK1 in relation to various clinical features. Additional studies and classifications are presented in Supplementary Fig. 5.

### 3.5. Transcription Factor Binding Site Analysis: ChIP-seq Data Exploration:

**Figure 6:**
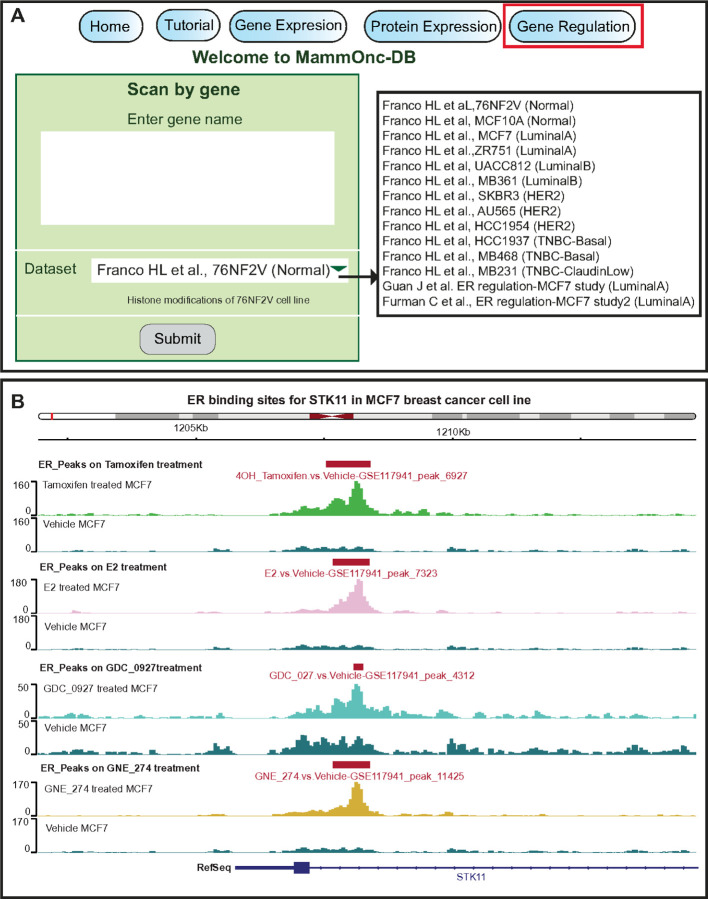
Gene regulation analysis functionalities. (A) Option for selecting gene of interest to investigate its regulation from studies available in MammOnc-DB. (B) IGV plot showing ER ligand binding in the region of *STK11*in MCF7 cell line is shown as an example here.

Processed ChIP-seq datasets are incorporated into MammOnc-DB to evaluate histone modifications and ER ligand treatment in different breast cancer BCa cell lines (GSE85158, GSE117941, and GSE178373). To facilitate interpretation, ChIP-seq results are presented in an interactive genome visualization format. Users can enter a specific gene and observe the binding of markers in either the promoter or gene body regions (Fig. 6A). Figure 6B shows a graphical representation of ChIP-seq results in MammOnc-DB. The figure displays the binding patterns of ER bound to different ligands (Tamoxifen, E2, GD 0927, and GNE 274) at *STK11* genomic locations in the MCF7 cell line, providing a visual depiction in the IGV.

Case studies have also been included and are available in Supplementary Document 1.

## 4. Discussion

Large-scale cancer omics data have been generated due to advancements in high-throughput technologies, including sequencing techniques and a reduction in the cost of sequencing. Omics data are critical for understanding the molecular changes and mechanisms underlying breast cancer development and progression, which can help to identify biomarkers and therapeutic targets. To maximize the utility of publicly available multi-omics data, there is a need to develop an easy-to-use web portal that enables researchers and clinicians to perform comprehensive analyses of these data and visualize them. Data collection, processing, and analysis require dedicated effort from experts in various fields, including pathology, computational biology, and statisticians.

We created MammOnc-DB platform to explicitly focuses on BCa-related omic data analysis and visualization. While our previous effort UALCAN provides pan-cancer data analysis ^66,67^, MammOnc-DB incorporates transcriptomics and proteomics data from various consortia and public repositories. This platform utilizes bulk RNA-seq, single-cell RNA-seq (scRNA-seq), ChIP-seq, and mass spectrometry (MS) data. Bulk RNA-seq provides a comprehensive view of gene expression patterns across tumor tissues, offering a broad understanding of the transcriptional landscape. Conversely, scRNA-seq explores the heterogeneity of cells, uncovering distinctive cell populations within tumors. This level of analysis is essential for identifying rare cell types, elucidating tumor progression, and mapping cellular lineage connections. Additionally, scRNA-seq data can unveil specific transcriptional profiles of individual cell types, which may be obscured in bulk RNA-seq data, facilitating a more accurate identification of potential therapeutic targets and biomarkers. ChIP-seq enables the discovery of DNA-protein interactions and epigenetic changes, shedding light on the regulatory processes governing gene expression. This approach is necessary for understanding the impact of transcription factors and other regulatory proteins on the advancement of BCa. In addition, MS investigations unveil the proteomic profile, outlining protein levels, modifications after translation, and interactions between proteins. By combining these sets of data, a holistic understanding of the molecular changes in BCa can be achieved.

Integrating multi-omics data in MammOnc-DB allows users to conduct *in-silico* analysis and validation of target genes that are specific to various tumor subgroups. This functionality facilitates hypothesis generation based on available data. Moreover, the platform serves as a tool for discovering new biomarkers crucial for early detection, prognosis, and prediction of responses to treatment. By analyzing pre- and post-treatment data, researchers and clinicians can identify markers that indicate therapy response, which could guide clinical decision-making. Incorporating gene expression, gene regulation, and protein data enhances the reliability of the identified biomarkers. Despite the advancements and the potential of MammOnc-DB, limitations should be acknowledged. Due to the lack of access to raw data, different normalization methods were present in the processed data, which could introduce variability and affect the comparability and interpretation of the results. Since MammOnc-DB relies on publicly available datasets, there is a potential for bias introduced by the selection and representation of these datasets.

We will maintain platform dynamics by integrating into MammOnc-DB additional molecular datasets, such as DNA copy number alterations, DNA methylation data from Illumina arrays, and information on transcription factor binding using ChIP-Seq data. Further, we will include additional datasets as they become available. Furthermore, we intend to analyze and include spatial transcriptomics data from public repositories. We expect to be responsive to user needs and suggestions when possible and will upgrade MammOnc-DB as appropriate. In summary, MammOnc-DB will serve as a valuable resource for BCa researchers and clinicians, enabling them to explore the diverse multi-omics data related to BCa and facilitating discoveries of BCa biomarkers and targets.

## Supplementary Material

Supplement 1

## Data Availability

The pre-processed data in this portal are available in the designated references. The underlying code for this portal is not publicly available but may be made available by the corresponding author to researchers on reasonable request.
