# Supplementary material for: MammOnc-DB, an integrative breast cancer data analysis platform for target discovery
Source: Res Sq. 2024 Sep 26:rs.3.rs-4926362. Preprint. [Version 1] doi: 10.21203/rs.3.rs-4926362/v1 (PMC11469468; doi:10.21203/rs.3.rs-4926362/v1)
Supplement: Supplement 1 [file NIHPPrs4926362v1-supplement-1.pdf]

Supplementary Files

This is a list of supplementary files associated with this preprint. Click to download.

- SupplTable1MammoncDatasets.pdf
- SupFig1.pdf
- SupFig2.pdf
- SupFig3.pdf
- SupFig4.pdf
- SupFig5.pdf
